# Supplementary material for: DNA Methylation-Specific Analysis of G Protein-Coupled Receptor-Related Genes in Pan-Cancer
Source: Genes (Basel). 2022 Jul 7;13(7):1213. doi: 10.3390/genes13071213 (PMC9320183; doi:10.3390/genes13071213)
Supplement: Supplementary file 1 [file genes-13-01213-s001.zip › Table S3.docx]

Table S3

| Cancer Type | The Number of Differential Sites | The Number of Differential Genes |
| --- | --- | --- |
| ACC | 327 | 262 |
| BLCA | 3791 | 1890 |
| BRCA | 6008 | 2844 |
| CESC | 1089 | 623 |
| CHOL | 3098 | 1847 |
| COAD | 8247 | 2843 |
| ESCA | 708 | 465 |
| HNSC | 5287 | 2368 |
| KICH | 2205 | 1522 |
| KIRC | 4639 | 2860 |
| KIRP | 2716 | 1662 |
| LIHC | 6545 | 2459 |
| LUAD | 1426 | 779 |
| LUSC | 6116 | 2829 |
| OV | 688 | 502 |
| PAAD | 26 | 25 |
| PCPG | 244 | 199 |
| PRAD | 4876 | 2567 |
| READ | 6962 | 2419 |
| SKCM | 999 | 629 |
| STAD | 1050 | 704 |
| TGCT | 707 | 580 |
| THCA | 856 | 700 |
| THYM | 21 | 17 |
| UCEC | 12129 | 4720 |
| UVM | 11801 | 5305 |
