# Supplementary figures and images for: DNA Methylation-Specific Analysis of G Protein-Coupled Receptor-Related Genes in Pan-Cancer
Source: Genes (Basel). 2022 Jul 7;13(7):1213. doi: 10.3390/genes13071213 (PMC9320183; doi:10.3390/genes13071213)

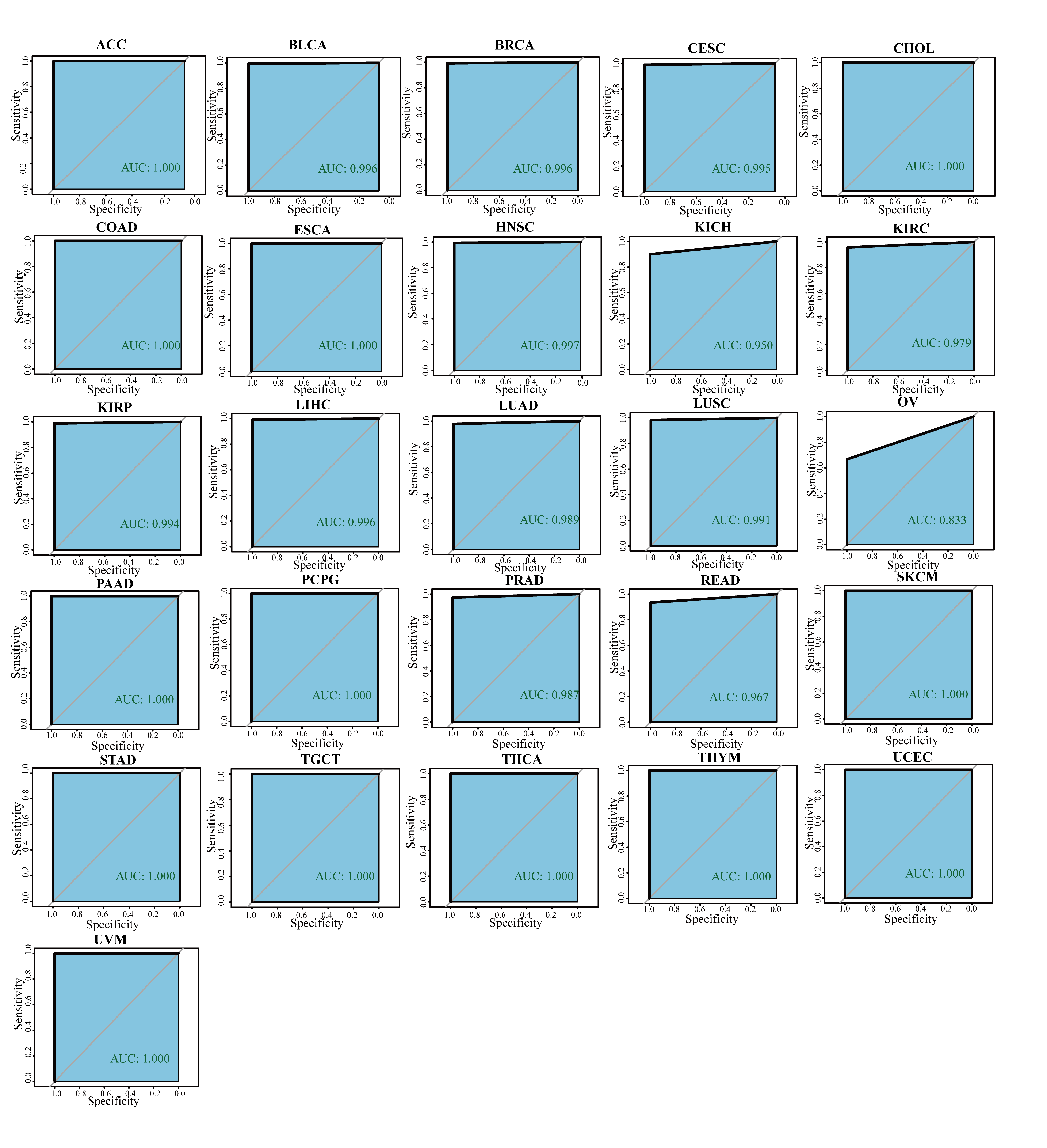

Supplement: Supplementary file 1 [file genes-13-01213-s001.zip › Figure S1.tif]
